# miRNA-targeted auxin nuclear signalling elements orchestrate flower fate and drought response in yellow lupine

**DOI:** 10.1038/s41598-025-22989-x

**Published:** 2025-11-10

**Authors:** Milena Kulasek, Paulina Glazińska

**Affiliations:** 1 Department of Genetics, Faculty of Biological and Veterinary Sciences, Nicolaus Copernicus University in Toruń, Toruń, Poland; 2 Centre for Modern Interdisciplinary Technologies, Nicolaus Copernicus University in Toruń, Toruń, Poland; 3 Department of Plant Physiology and Biotechnology, Faculty of Biological and Veterinary Sciences, Nicolaus Copernicus University in Toruń, Toruń, Poland

**Keywords:** IAA, *Lupinus luteus*, TIR1/AFB, ARF, miRNA, Drought, Developmental biology, Molecular biology, Plant sciences

## Abstract

Yellow lupine (*Lupinus luteus* L.) is a promising protein crop, but domestication has increased flower abscission. Flowers in higher whorls are more likely to abscise, providing a model to study factors influencing flower fate before abscission zone formation. We hypothesise that auxin localisation and miRNA-modulated signal transduction orchestrate flower development and abscission, particularly under drought. We investigated the indole-3-acetic acid (IAA) distribution in ovules and tested the effects of IAA, NPA (auxin transport inhibitor), and PCIB (auxin signalling blocker) on flower retention. Bioinformatic analyses of transcriptome data profiled miRNA-regulated auxin pathway elements (TIR1/AFBs, ARFs), with domain structures analysed for functional divergence. Selected miRNA-mRNA modules were analysed for their role in inflorescence drought response. IAA accumulation exhibited slight differences in upper pre-anthesis flowers. In the field experiment, PCIB treatment led to increased retention of generative organs, suggesting the vital role of auxin nuclear signalling in flower fate. Bioinformatic analyses identified miRNAs (MIR393, MIR160, MIR167, one novel) regulating *TIR1/AFB* and *ARF6*,*8*,*17*,*18*. qPCR revealed the differential expression of *AFB3*-, *ARF6*-, and *ARF18*-encoding transcripts and corresponding miR393, miR167, and miR160 during flower development and drought stress. However, miRNA-target expression lacked a strictly negative correlation, indicating other regulating players.

**Supplementary Information:**

The online version contains supplementary material available at 10.1038/s41598-025-22989-x.

## Introduction

The growing world population and shift towards a plant-based diet increase demand for high-quality plant protein. Responsible diet options benefit the environment, significantly reducing greenhouse gas emissions from animal farming^1^. Soybean has long been the primary non-animal protein source. Still, it has drawbacks: high allergenicity, trypsin inhibitors, and heavy dependence of Europe on soybean imports from transoceanic sources, increasing the carbon footprints. Consumer trust in soy has also declined due to GM concerns. These challenges are motivations to explore native European high-protein plant alternatives. One promising candidate is yellow lupine (*Lupinus luteus* L.), a pulse with ~ 40% seed protein and with the ability to form a symbiotic relationship with nitrogen-fixing bacteria. However, the domestication of yellow lupine a century ago inadvertently reduced yield due to an increased flower abscission rate, egregiously accelerated during drought^2^. Remarkably, yellow lupine is an excellent model for studying flower abscission, as the probability of shedding flowers rises with higher localisation along the inflorescence axis. Comparing flowers at extreme positions provides insights into the processes preceding the visible signs of abscission, such as abscission zone activation^3^.

Auxin is a key phytohormone regulating nearly all plant processes, including flower development^4–8^. It is produced locally^9^ or in specialised tissues and then transported to specific destinations, forming maxima and concentration gradients^10^. Auxin triggers cell division or elongation^11^, the two fundamental processes underlying plant development and stress response. The output results from activation of the nuclear auxin pathway, which involves perception through TRANSPORT INHIBITOR RESPONSE 1/AUXIN SIGNALING F-BOX (TIR1/AFB) receptors and transcriptional shift by auxin response factors (ARFs)^12^. ARFs dimerise through their C-terminal DNA-binding (DBD) domains^13^ and bind to symmetrically distributed Auxin Response Element (AuxRE) pairs^14^. At low auxin levels, Auxin/Indole-3-Acetic Acid (Aux/IAA) repressors bind to ARFs through their N-terminal PB1 domains and recruit TOPLESS (TPL) and TOPLESS-Related (TPR) proteins *via* an ethylene response factor (ERF)-associated amphiphilic repression (EAR) motif (LxLxL) within domain I, which leads to chromatin remodelling and gene repression^15^. When auxin binds to the TIR1/AFB receptor pocket formed by LRR loops 2, 12 and 14^16,17^, it enables stable binding of Aux/IAA. This complex binds to the Skp and Cullin proteins using the F-box domain, forming the active E3 SCF ligase complex (Skp, Cullin, F-box). Then, Aux/IAA is ubiquitinated and directed to proteasomal degradation^18^. Consequently, ARFs are released from repression and can regulate gene expression through their middle domains (reviewed in^19^:). Noteworthy, ARF diversity across cell types ensures context-specific auxin responses^20^.

Micro RNAs (miRNAs) are known to fine-tune this pathway, regulating *TIR1/AFB* and *ARF* expression. The canonical target of miR393 is *TIR1/AFB*. In *A. thaliana*, this miRNA accumulates during the leaf^21^ and root^22^ development, as well as in response to biotic^23^ and abiotic^24^ stresses, reducing auxin sensitivity. Conserved miRNA-ARF modules involved in plant generative development include *ARF6*/*8*-miRNA167^25,26^ and *ARF10*/*16*/*17*-miRNA160^27,28^. Interestingly, in *Arabidopsis* miR847 was found to trigger degradation of the *IAA28* gene transcript during the development of leaves, flowers, and lateral roots, as well as following exogenous NAA application^29^. However, our previous study in yellow lupine found no degradome-confirmed miRNA-Aux/IAA pair^30^, suggesting translation-level regulation or no involvement in flower development. New species-specific modules are still being discovered^30^.

The auxin-related miRNA-mRNA modules also mediate drought response in *Arabidopsis thaliana* and cereals^24,31^. One of the hallmark responses to this stress is lateral root growth. It is induced by abscisic acid, with simultaneous silencing of the auxin pathway *via* mir393-*TIR1*, miR160-*ARF10/16/17*, miR167-*ARF6/8*; and miR390/*TAS3*-ta-siRNA-ARF2/3/4 modules^31^. However, most studies focus on roots, and their role in flower abscission during drought stress remains unclear. Our previous high-throughput analysis in yellow lupine flowers identified auxin-related miRNA-mRNA modules acting during flower development^30^, leading us to hypothesise that these modules may be accelerated during drought stress, resulting in excessive flower abscission.

To test this, we have reviewed the previously published RNA-Seq data^30,32^ for degradome-confirmed miRNA-mRNA pairs involved in auxin nuclear signalling. Based on the structural and expression analyses, we selected three Ll-miRNAs and five targeted transcripts for expression profiling during drought stress across four stages of flower development and position on the inflorescence axis.

## Materials and methods

### Plant material

The seeds of yellow lupine (cv. Taper) were purchased from the Wiatrowo Plant Breeding Station (Poznańska Hodowla Plant Sp. z oo Tulce, Poland). Seeds were treated with a 3.5 ml/kg solution of Vitavax 200FS (Chemtura AgroSolutions, Middlebury, USA) to prevent fungal infections and then inoculated with Bradyrhizobium lupine cultures contained in Nitragin (BIOFOOD s.c., Walcz, Poland). Field crops were carried out in the Nicolaus Copernicus University experimental field in the Astronomical Observatory in Piwnice near Toruń, Poland.

**Fig. 1:**
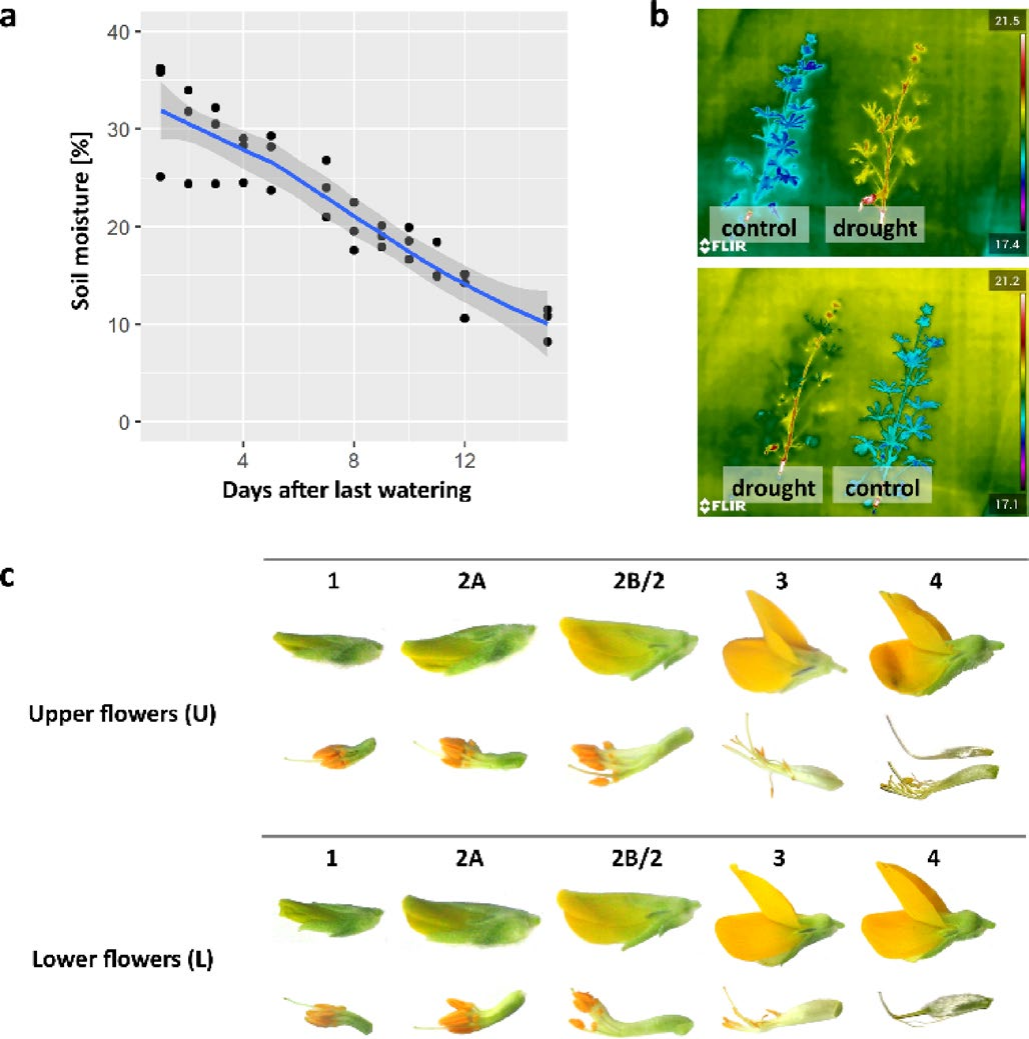
Control of the drought stress and sampled flower variants. **(a)** Plot showing soil moisture after stopping the watering. **(b)** Thermograms of plants used for drought experiment. **(c)** Yellow lupine flower development stages.

For the drought experiment, plants were grown in controlled conditions in the growth room. Drought was induced two weeks before the flowering and confirmed by soil moisture measurements and thermal imaging (Fig. 1a, b).

### Experimental variants

We collected flowers at stages one to four (1–4) growing on the highest whorls of the inflorescence (upper flowers, U1-4) and the lowest whorls (lower flowers, L1-4) (Fig. 1c). Stage 1 is characterised by closed green buds, where anthers and pistils are still elongating. Stage 2 comprises closed yellow buds just at the time of anther opening. Stage 3 flowers were in full anthesis. Stage 4 is open flowers one day after anthesis; in the upper whorl, it is arrested in development, and in the lower whorl, the pistil is enlarged. Stage 2 of yellow lupine flower development in the IAA localisation experiment was divided into two sub-stages: 2 A and 2B. In the remaining experiments, step 2 is equivalent to step 2B.

For field yield experiments, we selected 10–20 randomly dispersed plants at a very similar developmental stage of the inflorescence. In the “top” variant, flowers at the 2 A stage were located on the top whorl, while in the “bottom” variant, 2 A stage flowers were located on the bottom whorl. Treatments were conducted in the field between 9:00 and 10:00 a.m. using the control solution (0.05% Tween-20 in water), 50 µM IAA (in the control solution), 100 µM NPA (in the control solution), or 100 µM PCIB (in the control solution). Treated inflorescences were isolated from the rest of the plant with a plastic barrier and sprayed with the respective solutions. Plants were harvested at their technical maturity, and the mass and number of seeds were assessed for each plant.

### Immunolocation on sections of paraffin ovules

The entire fertile parts of the yellow lupine flowers were isolated and transferred to a pre-fixation solution (4% formaldehyde in 1x PBS). When all samples were isolated, they were transferred to a fixing solution (2% EDAC, 4% formaldehyde, 1x PBS) and incubated overnight at 4 °C. Then the samples were dehydrated in a series of ethanol solutions supplemented with 2% EDAC: 10%, 30%, 50%, for 1 h at 4 °C each, and 70% overnight at 4 °C. The dehydration was continued the next day with subsequent incubations in 80%, 96%, and two rounds of 100% ethanol, each lasting 1 h at 4 °C. The samples were subjected to a series of ethanol: butanol mixtures (3: 1, 1: 1, 1: 3, v: v), with concentrations changing every 15 min. Afterwards, they were placed in 100% butanol at room temperature. For embedding, the samples were heated to 56 °C using a heating block and the butanol was gradually replaced with mixtures of butanol and Paraplast Plus in the proportions of 3:1, 1:1, and 1:3 for 40 min. The samples were then transferred to 100% Paraplast and incubated overnight at 56 °C. The following day, the samples were placed in a mould filled with 100% Paraplast and left to solidify at room temperature. The blocks were stored at 4 ° C.

Using the HM355 S rotary microtome (MICROM Laborgeräte GmbH, Germany), the blocks were cut into 10 μm sections and dried at 40 °C overnight. They were then incubated at 60 °C for 15 min and dewaxed and hydrated with the following washings: 3 × 100% xylene after 10 min, 100% xylene + 100% ethanol (1:1) for 2 min, 2 × 100% ethanol after 2 min, a series of aqueous 96%, 85%, 70%, 50%, 30% ethanol solutions (5 min each) and finally water 2 × 5 min. For antigen retrieval, slides were incubated in 95 °C citrate buffer (10 mM sodium citrate, 0.05% Tween-20, pH 6.0) for 2 min and washed three times in 1x PBS for 5 min each. Slides were blocked in 3% Fraction V BSA in 1x PBS in a humid chamber at 4 °C for 1 h, then incubated at 4 °C overnight in a solution of rabbit anti-IAA primary antibodies (Agrisera, Cat. No. AS06 193) 1:60, made in 1% Fraction V BSA, 1x PBS. Then, the slides were rinsed 1x with PBS, incubated for 10 min in 0.3% H_2_O_2_ with 1x PBS to inactivate endogenous peroxidases, and rinsed twice with 1x PBS for 5 min each. A solution of anti-rabbit secondary antibodies labelled with HRP (Agrisera, cat. no. AS10 833), prepared in 1% fraction V BSA, 1x PBS, was applied to the slides in a ratio of 1:150 and incubated overnight in a humid chamber at 4 °C. Then the samples were rinsed three times and visualised with 0.1% DAB solution (in 10 mM Na_2_HPO_4_ with 0.3% H_2_O_2_). Negative controls were incubated in 1% Fraction V BSA, 1x PBS instead of primary antibody solution.

### Data acquisition and filtering

Data concerning yellow lupine flower transcriptomes (RNA and predicted protein sequences, annotations, and expression) were downloaded from LuluDB database^32^. The sequences annotated as *TIR1*, *AFB*, *Aux/IAA* or *ARF* containing only complete open reading frames were extracted using Python 3 ‘pandas’. Small RNA-Seq and degradome-Seq data (sequences, miRBase annotations, expression, targets) were also downloaded from LuluDB database^32^ and filtered using Python 3 ‘pandas’.

Based on degradome-Seq data for yellow lupine flowers^30,32^ and using ‘circos’ R package, we created a plot depicting multi-target regulation of ARF transcripts by sRNA. Logos of miRNA sequences and their target sites were created with the WebLogo tool (https://weblogo.berkeley.edu/logo.cgi)^33^. We have also predicted the cleavage site for each identified miRNA-mRNA pair based on degradome data obtained from our previously published data^30,32^.

### Identification of miRNA precursors

We have identified the potential candidates for miRNA precursors by applying the following procedure. With Python 3 script we have filtered out the sequences which did not contain the sequences of mature miRNAs. We predicted secondary structure of RNAs with RNAfold and removed from the dataset the transcripts, in which the miRNAs were located outside the stem of the stem-loop structure within this RNA.

### Detailed review of predicted proteins

#### Structural analysis of miRNA-targeted TIR1/AFB nuclear auxin receptors

The tertiary structure of the AtTIR1 protein and its IAA binding pocket were visualised using data available in the Protein Data Bank (PDB AtTIR1 ID: 2P1P^16^ accessed through rcsb.org^34^ using the NGL viewer tool^35^. The tertiary structures of the putative TIR1/AFB family proteins were modelled using the I-TASSER tool (https://zhanggroup.org/I-TASSER/^36^). To prepare the proteins for molecular docking, the AtTIR1 protein structure was first converted to.pdb format using Pymol 2.5.2 and then to.pdbqt format using AutoDockTools 1.5.7. The visualisation of the protein structures was carried out using the Chimera 1.15 program. Molecular docking of the TIR1/AFB family proteins identified in yellow lupine flowers was performed using AutoDockTools 1.5.7 and PyMol 2.5.2. Prior to docking, water molecules were removed from the protein structure, polar hydrogens were added, and Kolmar charges were applied. The processed structures were saved in the.pdbqt format. The docking zone (grid) was defined to cover the area of the LRR ring. AutoDock Vina was used for the docking process with the following parameters: 50 repetitions of the algorithm and a population size of 300.

#### Structural analysis of miRNA-targeted ARF transcription factors

Sequences and domain coordinates (Pfam domain annotation) of putative ARF proteins were extracted from files downloaded from the LuluDB database^32^. The alignments showing key sequences were visualised using the ‘ggmsa’ R package. The amino acid composition of Middle Domains (MD) was calculated for each identified ARF with MEGA 11.0.10 software^37^ and plotted using the ‘ggplot2’ R package. Phox and Bem1 (PB1) sequences were aligned using MEGA 11.0.10^37^ and visualised using the ‘ggmsa’ R package. The consensus sequence was submitted to the PSSpred tool (Protein Secondary Structure prediction, https://zhanggroup.org/PSSpred/)^38^ for secondary structure analysis.

#### Phylogenetic analysis of miRNA-targeted TIR1/AFBs and ARFs

The amino acid sequences of TIR1s, AFBs, and ARFs homologues in other plant species were retrieved from the NCBI Genes database for phylogenetic analysis. Sequence alignment was performed using the MUSCLE algorithm within MEGA 11.0.10 software^37^. Subsequently, a phylogenetic tree was constructed using the maximum likelihood (ML) method on the JTT model with 100 bootstrap replications. The resulting tree was then visualised using the Interactive Tree of Life (iTOL) v.5 online tool^39^.

### Expression of MiRNAs and targeted transcripts from RNA-Seq data

Data concerning *TIR1/AFB* and *ARF* expression in yellow lupine flowers were downloaded from LuluDB^32^. The levels of transcript accumulation were shown in the Fragments Per Kilobase Million (FPKM) unit, which means the fragment counts for a given transcript were normalised to million reads of the total RNA fragments in the sample and the length of the transcript in kilobases. We have plotted these data using the ComplexHeatmap R package.

Data concerning the expression of miRNAs targeting transcripts that encode TIR1/AFBs, and ARFs in yellow lupine flowers were downloaded from LuluDB^32^. Accumulation of these regulatory molecules in samples was expressed in the Reads Per Million (RPM) unit, which means the reads concerning a given miRNA were normalised to million reads of the total sRNA reads in the sample. We have plotted these data using the ComplexHeatmap R package.

### Expression levels of *LlTIR1*/*AFB*, *ARFs* and regulatory miRNA during drought

Drought was induced by stopping watering pots with yellow lupine. The loss of water content was checked by infrared thermography. Plants with lower temperatures did not undergo the stress (control watered pots), and those with similar temperatures to the environment underwent the stress. We sampled the flowers from upper and lower whorls and homogenised them in liquid nitrogen. We used miRNeasy Mini kit (QIAGEN, Germany) following the procedure variant with on-column DNA digestion for total RNA isolation. Reverse transcription for transcripts and miRNAs was performed differently. In the first case, we used NG dART kit (EurX, Gdansk, Poland). For sRNA, we used a method with hairpin primers developed by Kramer et al. [196] and modified by Varkonyi-Gasic et al. [197] and our group^30,32^. The list of primers used for reverse transcription and qPCR is shown in Table S7.

We performed qPCR analysis of miRNAs and their targets according to^30,32^. Each experiment consisted of three biological and technical replicates. The relative expression levels were calculated using the 2−∆∆Ct method, and the data were normalised to the CT values for the *LlActin* reference gene (according to^30,32^). All primer sequences are listed in Table S7.

**Ethical approval**. This study was conducted in accordance with all relevant institutional, national, and international guidelines for plant research, including the IUCN Policy Statement on Research Involving Species at Risk of Extinction and the Convention on the Trade in Endangered Species of Wild Fauna and Flora. Yellow lupine (*Lupinus luteus*) is a cultivated crop and is not classified as an endangered or protected species. No special permits were required for the collection or use of plant material.

## Results

### Auxin distribution pattern and effects of disruption of auxin functioning in yellow lupine flowers

**Fig. 2:**
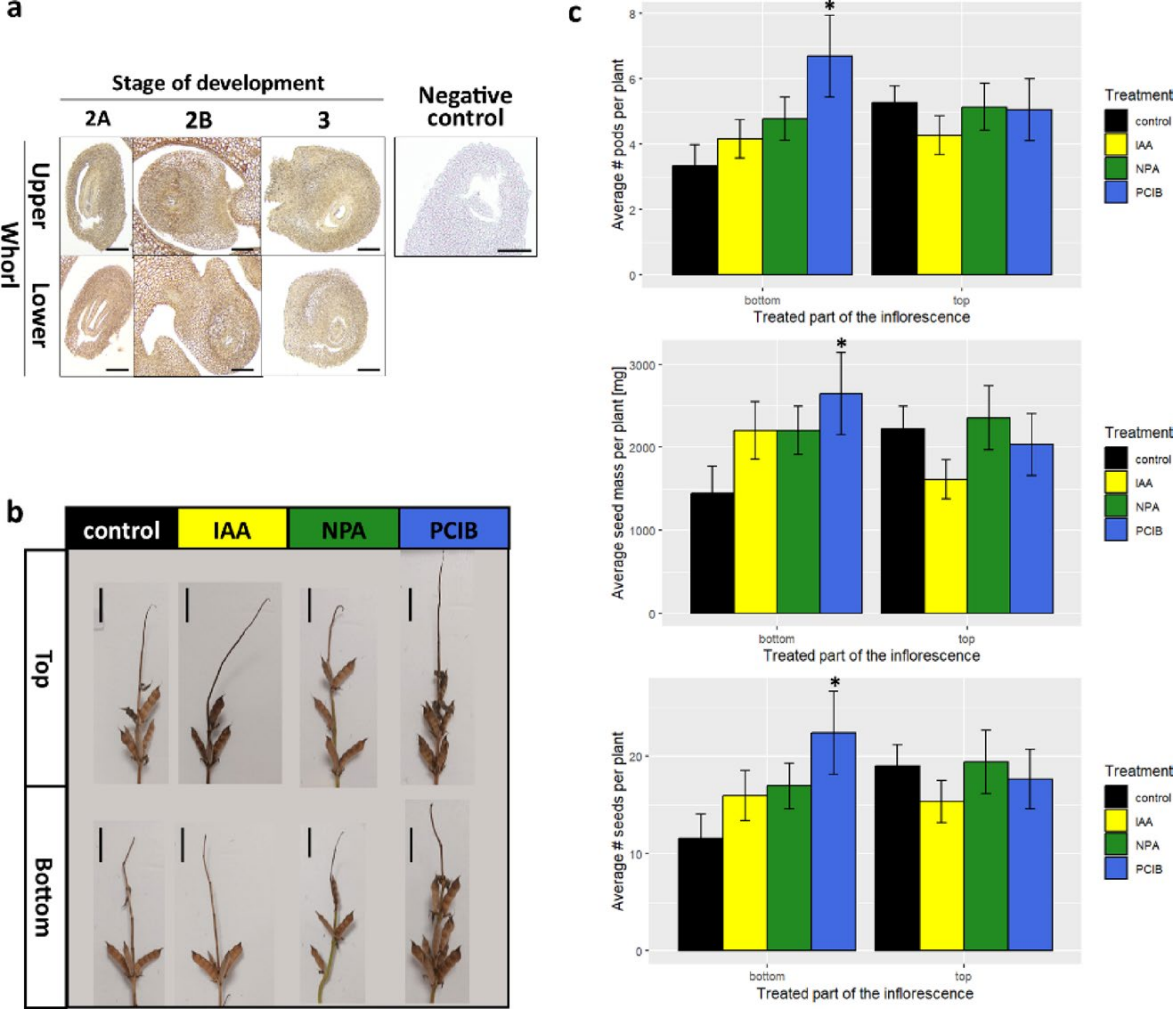
Results of field experiment focused on auxin in yellow lupine flowers. **(a)** Localisation of indole-3-acetic acid (IAA) in paraffin sections of ovules isolated from developing yellow lupine flowers. Brown coloration indicates IAA accumulation. The scale bar represents 0.2 mm. **(b)** Representative images of plants harvested at the technical maturity stage after treatments with the mock solution (control), 100 µM indole-3-acetic acid (IAA), 100 µM N-1-naphthylphthalamic acid (NPA), and 100 µM p-chlorophenoxyisobutyric acid (PCIB). **(c)** Yield of plants subjected to mock solution, IAA, NPA, or PCIB treatments. Asterisks indicate significant differences (*p* < 0.05, t test) compared to the control.

To gain insight into the role of auxin in lupine flower fate, we localised this hormone in ovules at different developmental stages and from opposite whorls (Fig. 1c). The brown colouration indicates the presence of auxin. Regardless of the flower position on the inflorescence axis, IAA accumulates throughout ovules, with the more intensive signal within the inner integuments. Intriguingly, in ovules isolated from the upper stage 2B flowers, the signal was slightly stronger in the outer integuments (Fig. 2a).

Next, we performed a field experiment to examine the importance of auxin distribution or nuclear signalling in generative organ fate. We have treated lupine inflorescences when the flowers at the lowest (‘bottom’) or highest (‘ top’) whorl were at the 2 A stage of development (Fig. 1c). The plants treated with 100 µM, p-Chlorophenoxyisobutyric acid (PCIB, which blocks the auxin signal transduction pathway^40^ at the bottom part of the inflorescence showed higher retention of generative organs and, consequently, higher mass and number of seeds per plant (Fig. 2b, c). 100 µM indole-3-acetic acid (IAA), 100 µM N-1-naphthylphthalamic acid (NPA, which blocks auxin transport^41^ treatments had no effect on the studied yield parameters (Fig. 2b, c).

### Identification of miRNA-regulated elements of auxin signal transduction pathway

**Fig. 3:**
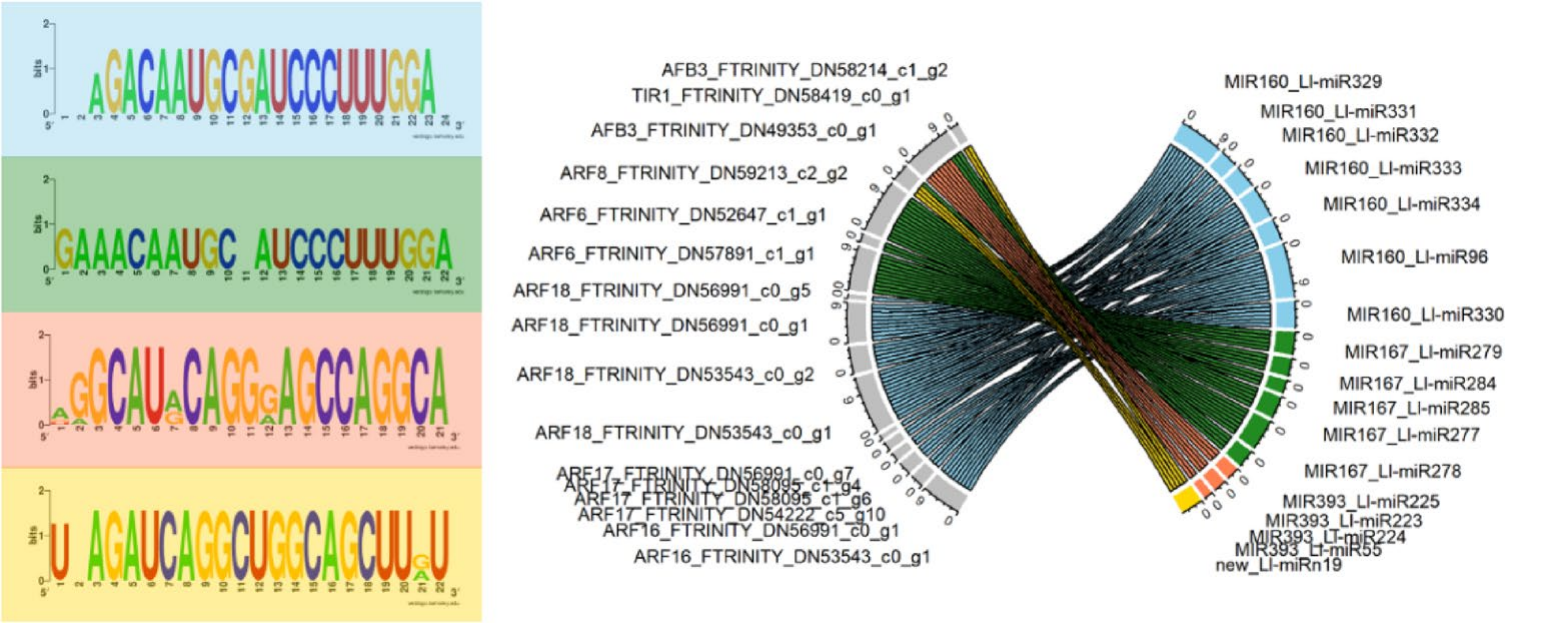
miRNA-mediated regulation of transcripts annotated as *TIR1/AFB* and *ARF* in developing yellow lupine flowers, as revealed by degradome sequencing. The left side displays logos of target sequences. The right side features a Circos plot illustrating the complex, multi-directional regulation of *TIR1/AFB* and *ARF* expression by miRNAs from the MIR160, MIR167, and MIR393 families. Details about the cleavage sites and T-plots are presented in Figures S1−3.

The subsequent objective was to explore the auxin-related miRNAs-mRNA modules in yellow lupine flowers through an integrated analysis of RNA-Seq, sRNA-Seq, and degradome-Seq data from open-access LuluDB database^32^. We filtered the data to obtain a dataset containing only information about transcripts with complete open reading frames and that are annotated as LlTIR1/LlAFBs, Aux/IAAs, and LlARFs, with a record in degradome data. This analysis revealed the conserved regulatory modules: *MIR393-TIR1/AFB3*, *MIR167-ARF6/8* and *MIR160-ARF17/18* and one new miRNA targeting transcripts that encode LlTIR1/LlAFB2 (Fig. 3). However, this miRNA is highly similar to Ll-miR393 with a single-nucleotide difference at the 5’ end, suggesting it may be a member of this family (Table S1).

We also identified potential precursor transcripts for these miRNAs. For the MIR160 family, eight transcripts were found (Figure S4); for MIR167, three transcripts (Figure S6); and MIR393, ten transcripts (Figure S8). Only nine miRNA–precursor pairs exhibited a Spearman correlation coefficient greater than 0.5 (Table S3; Figures S5, S7, and S9), suggesting that additional factors substantially influence the expression and stability of these transcripts. Moreover, the efficiency of processing these RNAs into mature miRNAs may vary depending on mismatch positions and nucleotide composition within the hairpin structure^42^, as well as the presence of R-loops^43^. Therefore, in this study, to avoid excessive complexity, we focused exclusively on mature miRNAs, which are the final regulators of post-transcriptional gene expression.

### Manual revision of elements of miRNA-targeted auxin signal transduction pathway in yellow lupine flowers

#### miRNA-targeted TIR1/AFB family

**Fig. 4:**
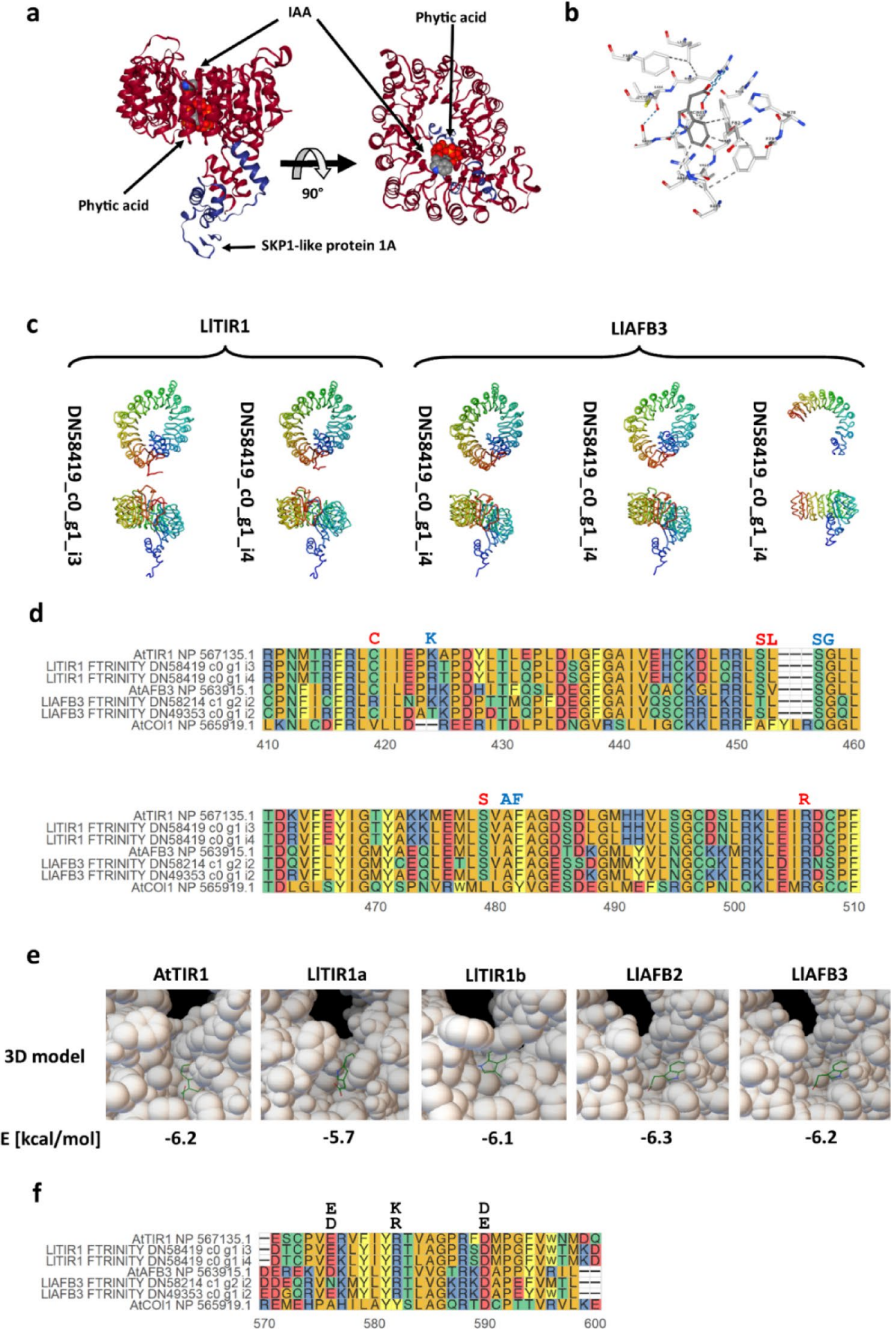
Analysis of the primary and tertiary structure of proteins from the TIR1/AFB family identified in yellow lupine flowers. **(a)** The structure of AtTIR1 in *Arabidopsis thaliana* and the positions of its ligands: phytic acid and indole acetic acid (IAA), depicted on the basis of data obtained from the PDB database (2P1P) using the built-in NGL viewer tool. **(b)** IAA binding pocket of AtTIR1 visualised with NGL viewer. **(c)** Structural models of TIR1/AFB family proteins identified in yellow lupine obtained with I-TASSER and visualised with Chimera 1.15 software. **(d)** MSA analysis of amino acid sequences of auxin-binding pockets in TIR1/AFB family proteins identified in yellow lupine (Ll) and their homologues in *A. thaliana* (At). Jasmonic acid receptor (COI1) sequences constituted an external group for the performed analyses. The letters above the comparison represent amino acids crucial for forming the IAA binding pocket; red font indicates they are critical for ligand selectivity. **(e)** The 3D models of IAA binding pockets of analysed proteins with docked IAA. **(f)** MSA analysis of amino acid sequences of predicted AC domains in yellow lupine (Ll) and their homologues in *A. thaliana* (At). The letters above the comparison are amino acids critical for AC activity.

The initial filtration based on the TIR1/AFB family annotation and record in degradomes resulted in the identification of 11 transcripts, six of which encoded putative F-box-containing proteins (Table S2). SMART analysis revealed that only 4 had high-quality F-box domains. Further analysis confirmed whether the C-terminal part rich in Leucine-Rich Repeats (LRRs) formed a closed ring and contained IAA-binding pockets. Structural analyses, including 3D testing and in silico docking, were performed using *Arabidopsis* TIR1 reference structures (Figs. 4a, b). The FTRINITY_DN49353_c0_g1_i4 transcript was shown to encode an incomplete protein in which the repeated LRR motifs do not form a closed ring containing the auxin docking pocket (Fig. 4c) and was therefore excluded from further analyses. A complete three-dimensional structure characterised the remaining identified receptors (Fig. 4c).

We examined the presence of key amino acids in the predicted proteins that are known to form a functional IAA binding pocket. According to AtTIR1 crystallographic studies, the pocket involved in the proper orientation of the ligand is formed by Lys-410, Ser-440, Gly-441, Ala-464 and Phe-465, while Cys-405, Ser-438, Leu-439, Ser-440, Ser-462 and Arg-489 are responsible for ligand selection^44^. The multiple sequence alignment (msa) showed that LlTIR1a, LlTIR1b and LlAFB2 contain evolutionarily conserved amino acids interacting with IAA (Fig. 4d). Only the LlAFB3 sequence displayed differences in this region; however, the *in silico* docking experiment suggested its affinity to auxin remained unchanged (Fig. 4e).

Additionally, we have predicted the C-terminal ATP cyclase (AC) domain, as recent evidence indicate that TIR1/AFB proteins in *A. thaliana* possess the activity of ATP cyclase (AC). In *Arabidopsis*, mutation in any of three key amino acids within the C-terminal AC domain results in not only the reduction in cAMP production but also in the delay in nuclear response to auxin^45^. In LlAFB3, the substitution of polar asparagine residue in the first critical position with the negatively charged aspartic and glutamic acid residues (Fig. 4f) likely leads to a delayed auxin signal transduction *via* this receptor. This hypothesis needs further verification in the future.

#### miRNA-targeted ARF family

**Fig. 5:**
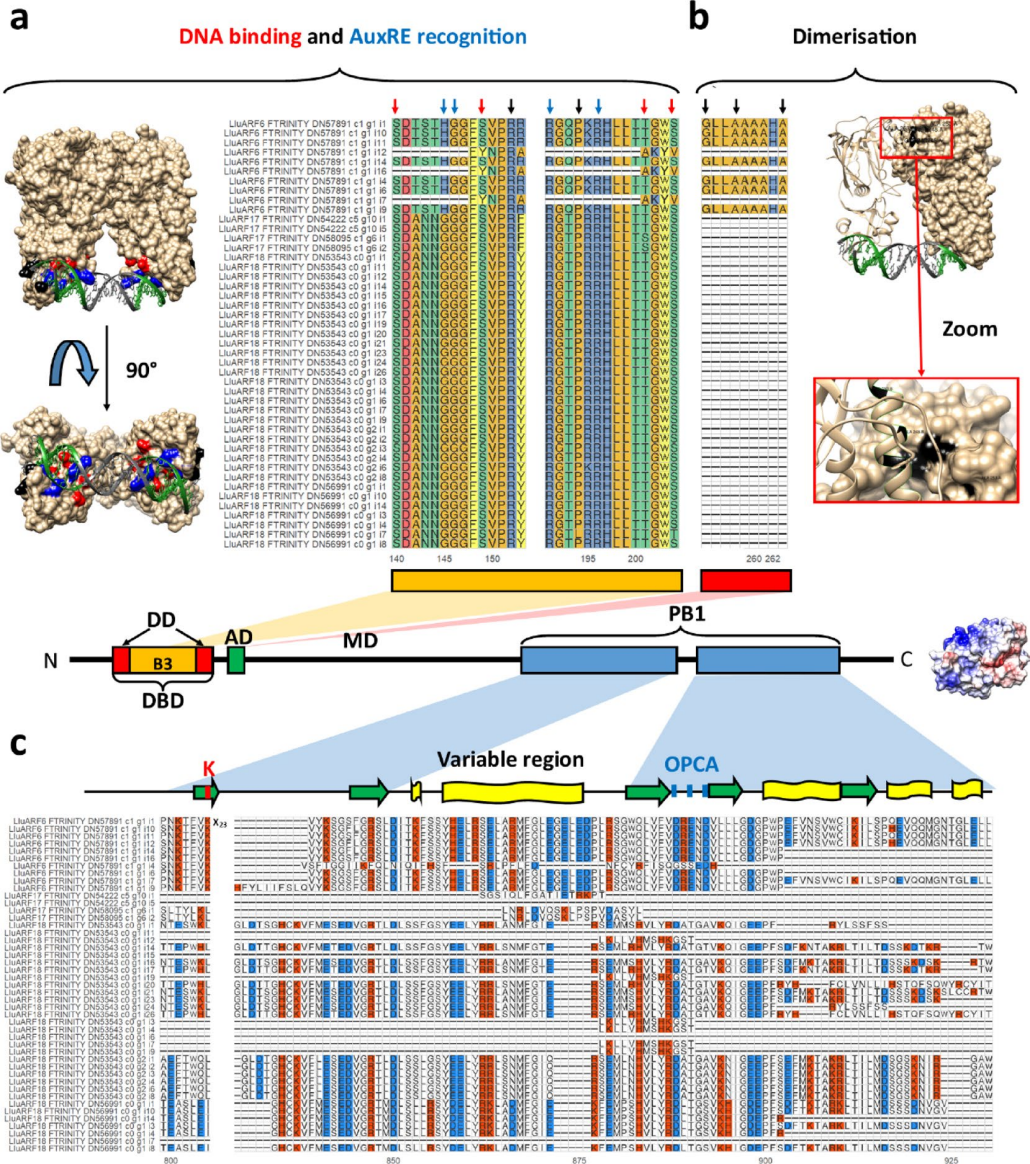
In-depth analysis of the DNA Binding Domain (DBD), and Phox and Bem 1 domain (PB1) within predicted LlARF proteins identified in yellow lupine flowers. **(a**,** b)** multiple sequence alignment of key motifs within DBD, responsible for **(a)** DNA binding and recognition of AuxRE, **(b)** dimerisation with other ARF proteins. Red arrows indicate amino acids involved in DNA binding, blue arrows indicate amino acids involved in AuxRE sequence recognition, and black arrows indicate the remaining amino acids that significantly contribute to DBD function. 3D models of AtARF1 DBD in complex with promoter-like sequence ER7 were downloaded from the RCSB PDB database (4LDX) and visualised in Chimera 1.15. **(c)** Multiple sequence alignment of PB1 domain. Red and blue shadings indicate positively and negatively charged amino acid residues, respectively. The 3D model of LlARF6a PB1 domain was obtained by 2D sequence modelling with I-TASSER (https://zhanggroup.org/I-TASSER/) and visualised using Chimera 1.15. Sequences were aligned using MEGA 11.0.10 and visualised using ‘ggmsa’ R package. Different colours were used to highlight the chemical properties of amino acid residues.

**Fig. 6:**
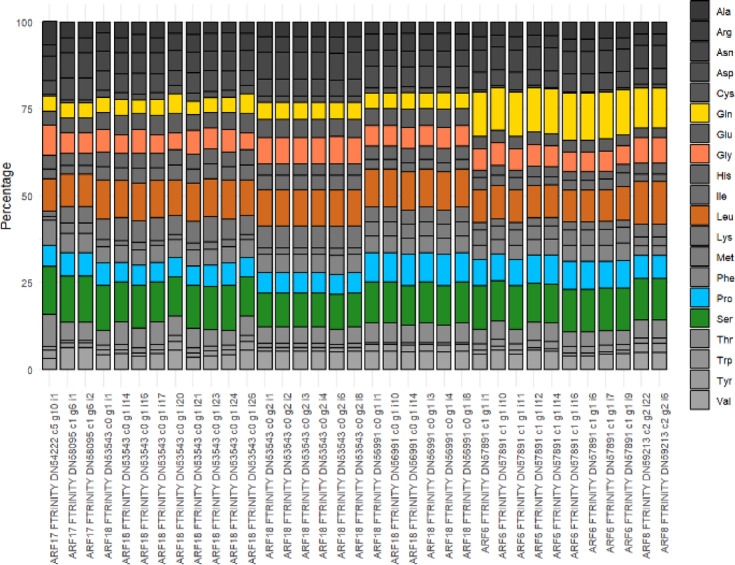
Amino acid composition of middle domains of LlARFs annotated as transcription activators or repressors.

**Fig. 7:**
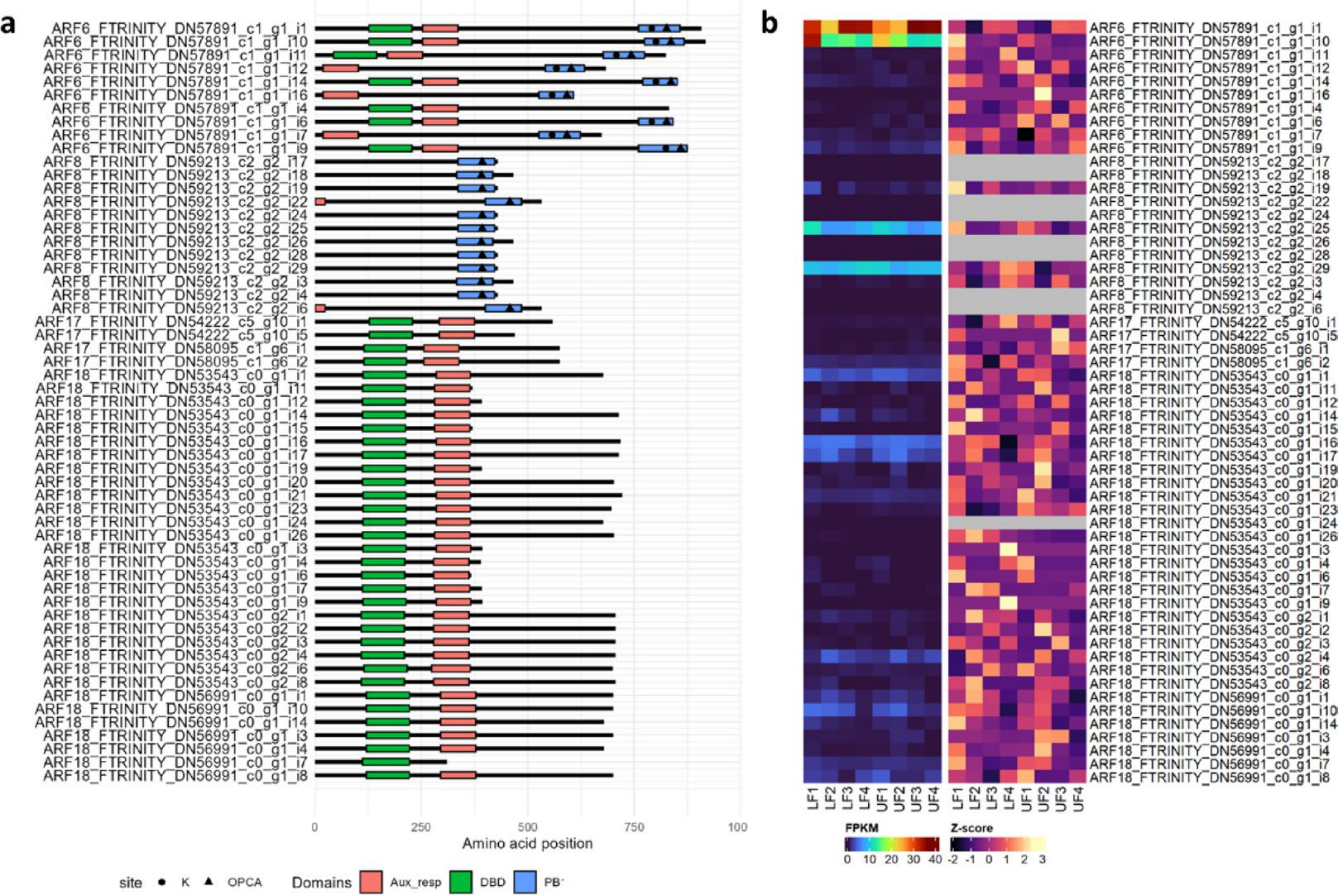
**(a)** Domain compositions of putative LlARF proteins encoded by miRNA-targeted transcripts. **(b)** Expression profiles of these proteins in yellow lupine flowers across developmental stages. Both raw and Z-score normalised expression data are shown.

Next, we explored sequences of key ARF domains (Figs. 5, 6 and 7, Table S4-5). A total of 158 *LlARF* transcripts containing complete open reading frames were initially selected based on BLASTX search results in the LuluDB database. We rejected transcripts that encoded proteins devoid of Pfam-predicted DBD or PB1 domains. This preliminary analysis allowed us to identify 16 *LlARF* genes transcribed into 69 transcripts.

Next, we focused on the amino acid sequences of key ARF domains: DBD, MD and PB1. The DBD domain comprises the middle DNA-binding domain (DB3), responsible for recognition and binding to the AuxRE motif, and two flanking parts of the DD domain, responsible for the dimerisation of ARF proteins. Most of the DBD-containing ARFs exhibited all the key amino acids necessary for DNA interaction and selectivity for the AuxRE element (Fig. 5a). Three transcript isoforms encoding LlARF6 (FTRINITY_DN57891_c1_g1_i7, _i12, and _i16) lack entire DBD, and all identified as LlARF17 and ARF18 lack crucial part of DD, which may affect their ability to homodimerise (Fig. 5b). Interestingly, all LlARF17 and LlARF18 had a glycine substitution for the histidine in the SxxxxHGxxSxxR motif, which is crucial for recognising the TGTCNN sequence. This observation suggests that these proteins may exhibit variations in selectivity and/or affinity for the AuxRE element (Fig. 5a).

Next, we investigated the distribution of negatively and positively charged amino acid residues within PB1 domain (Fig. 5c), responsible for dimerisation with other ARFs and Aux/IAAs^46^. Critical lysine (K) in the canonical position at one “face” (motif I) and acidic OPCA (motif II) are present only in the predicted LlARF6s, except for FTRINITY_DN57891_c1_g1_i4-encoded ARF6 isoform, which is missing the OPCA. None of the predicted miRNA-targeted LlARF8, ARF17 or ARF18 harbours the PB1 domain (Figs. 5c and 7, Table S4).

Next, we analysed the amino acid composition of MD domains, which determine whether ARFs function as transcriptional activators or repressors and are located between the DBD and PB1 domains. Based on this analysis, we identified potential transcriptional activators LlARF6 and LlARF8, all of which contained MDs enriched in glutamine (Gln), serine (Ser), and leucine (Leu) (Fig. 6). In *Arabidopsis* ARFs, MDs with this composition are known to function as activation domains^13,47^. Conversely, the MDs in other LlARFs were enriched in serine (Ser), leucine (Leu), proline (Pro), and glycine (Gly), resembling the repression domains characteristic of *Arabidopsis* ARF repressors^13,47^ (Fig. 6). The remaining ARFs identified in this study lack the middle domain and may instead be involved in either masking the AuxRE elements if composed exclusively of DBDs or terminating the ARF-Aux/IAA polymerisation if composed solely of PB1 domains.

The comprehensive analysis of domain sequences led to a reduction in the LlARF landscape, resulting in 14 genes and 56 transcripts (Fig. 7a, Table S2). Out of them, ARF6-encoding FTRINITY_DN57891_c1_g1_i1 and _i10 transcripts showed the highest expression (Fig. 7b), and they show the complete set of domains (Fig. 7a). Thus, these ARFs seem to be predominantly involved in the auxin signal transduction pathway in lupine flowers. Also, ARF8-encoding FTRINITY_DN59213_c2_g2_i25 and _29 as well as ARF18-encoding FTRINITY_DN53543_c0_g1_i1, _i16, _i17, FTRINITY_DN53543_c0_g2_i4, all FTRINITY_DN56991_c0_g1 show moderate expression. However, all the ARF8 proteins mentioned here contain only type I PB1 domains (characterised by the OPCA motif but lacking canonical lysine), suggesting that they primarily modulate the dynamics of ARF6-Aux/IAA polymerisation rather than functioning as transcription factors. ARF18s lack PB1 domains entirely, which is consistent with the expected characteristics of this subfamily.

#### Evolutionary conservation of miRNA-targeted TIR1/AFB and ARF families

**Fig. 8:**
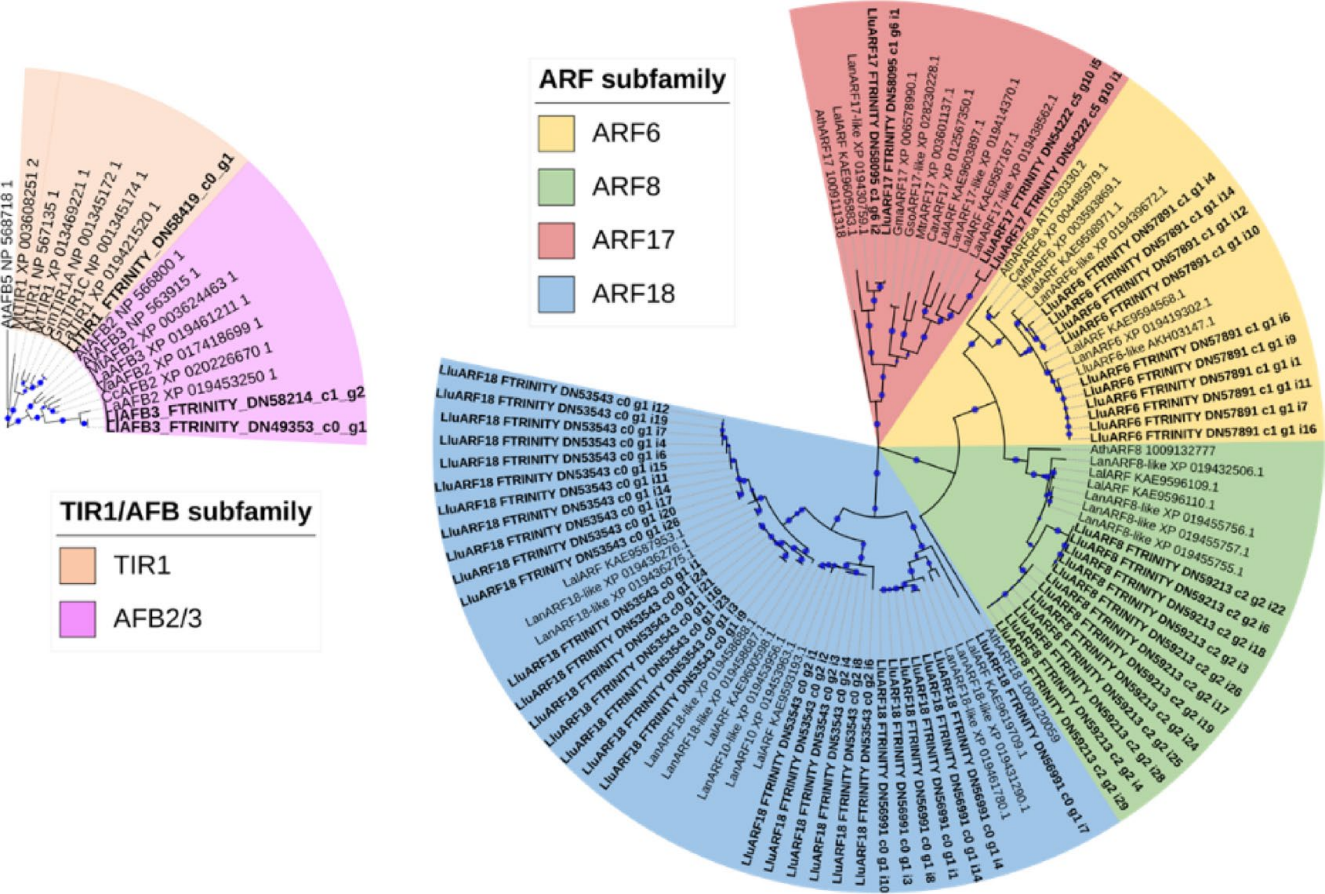
Phylogenetic tree constructed using amino acid sequences of TIR1/AFB and ARF proteins identified in yellow lupine flowers alongside with homologous proteins from *Arabidopsis thaliana* (Ath), *Lupinus angustifolius* (Lan), *Lupinus albus* (Lal), *Glycine max* (Gma), *Glycine soja* (Gso), *Medicago truncatula* (Mtr), *Cicer arietinum* (Car), *Pisum sativum* (Psa), *Trifolium pratense* (Tpr). The NCBI database identifiers are indicated on the leaf labels. For improved readability, branches lacking *L. luteus* sequences were removed from the tree.

To conduct a phylogenetic analysis of the selected putative TIR1/AFB and ARF proteins, we obtained amino acid sequences of homologues of these proteins from selected legumes and *Arabidopsis thaliana* from the NCBI database. Next, we constructed an ML tree using MEGA 11.0.11 software. For the vast majority of the analysed protein sequences, their closest homologues were found in *L. angustifolius*, and for the rest, *L. albus*. As anticipated, ARF amino acid sequences analysed in this study clustered into two distinct clades^48^: clade A, comprising ARF transcription activators (ARF6/8) and clade C, which includes the oldest ARFs that evolved before the emergence of land plants, such as the identified LlARF17. LlARF18 was also grouped within clade C, indicating its closer relationship with the ancient ARF17 subfamily. Notably, we did not identify *LlARF1/2/3/4* among floral miRNA-targeted genes, resulting in the absence of clade B from the phylogenetic tree (Fig. 8).

### Expression of *LlTIR1/LlAFBs* and *LlARFs* in yellow lupine flowers during drought

**Fig. 9:**
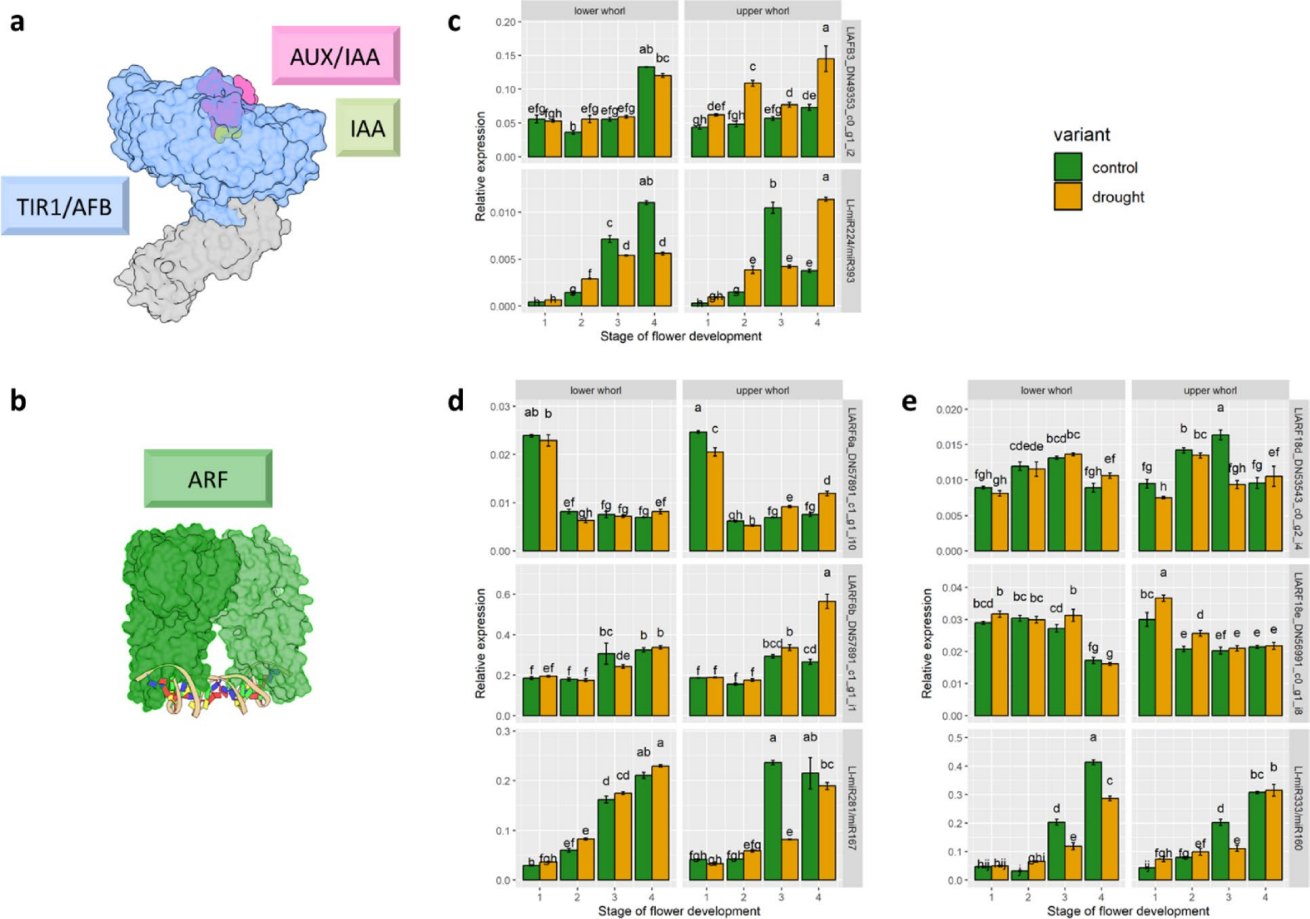
Expression of auxin-related miRNA-mRNA modules in yellow lupine flowers during drought. **(a**,** b)** The models generated using ChimeraX 1.7.1, based on PDB structures of **(a)** TIR1 (PDB ID: 2P1Q) and **(b)** ARF1 (PDB ID: 4LDQ). **(c-e)** Expression of selected elements from the **(c)** AFB3/miR393, **(d)** ARF6/miR167, and **(e)** ARF18/miR160 modules in yellow lupine flowers under control conditions and during drought. For **(c-e)** statistical analysis was performed using ANOVA followed by Tukey’s post-hoc test. Different letters indicate statistically significant differences (*p* < 0.05).

Recognising that the process of flower abscission is accelerated during drought in yellow lupine, we conducted an expression analysis of selected miRNA-mRNA modules in flowers under this stress, focusing on transcripts encoding TIR1/AFB receptors (Fig. 9a) and ARF transcription factors (Fig. 9b). We chose miRNA-target pairs with the highest expression and dynamics levels shown in RNA-Seq data: FTRINITY_DN49353_c0_g1_i2 (encoding LlAFB3) and Ll-miR224/miR393, FTRINITY_DN57891_c1_g1_i10, FTRINITY_ DN57891_c1_g1_i1 (encoding LlARF6a and b, respectively) and LlmiR281/miR167, FTRINITY_DN53543_c0_g2_i4, FTRINITY_DN56991_c0_g1_i8 (encoding LlARF18d and e, respectively) and Ll-miR329/miR160.

Our qPCR analysis shows that the LlAFB3-encoding transcript is primarily expressed in the fully open flowers (Fig. 9c), which suggests that floral tissues are most sensitive to auxin at this stage. It is worth noting that fully opened flowers located at the top whorl show higher *AFB3* expression, which suggests that modulation of IAA sensitivity may be one of the means for designating the flower fate. Transcript isoforms i10 and i1 of *LlARF6* gene seem to play distinct roles in flower development, as the first mentioned is primarily accumulated in the closed green buds and the latter in fully opened flowers (Fig. 9d). Interestingly, the two analysed transcripts coding for LlARF18d and LlARF18e show opposite differences in expression between the highest and lowest whorls: the first is more accumulated in U2-3, and the latter in L2-3 (Fig. 9e). This suggests their distinct roles in regulating flower abscission. The expression patterns of analysed miRNAs and their targets show no correlation (Fig. 9c-e). The studied miRNAs primarily accumulate in flowers in full anthesis, with higher levels in U3 in the case of Ll-miR224/miR393 (Fig. 9c) and Ll-miR281/miR167 (Fig. 9d), and in U2 in the case of Ll-miR329/miR160 (Fig. 9e). These results strongly suggest that miRNA-directed post-transcriptional regulation of these genes predominantly occurs in open buds, potentially playing a significant role in determining flower fate. Furthermore, the studied miRNAs appear to target multiple mRNAs.

In drought-stressed plants, the transcript encoding AFB3 exhibited higher abundance in upper whorls both before and after abscission (U2 and U4). The accumulation of Ll-miR224/miR393 did not correlate with this pattern; however, it was significantly higher in U4 flowers and lower in L3-4 and U3 compared to the control (Fig. 9c). The expression of the LlARF6-encoding FTRINITY_DN57891_c1_g1_i10 transcript was significantly lower in L2 and U1 and higher in U3-4 under drought conditions compared to the control. In contrast, the FTRINITY_DN57891_c1_g1_i1 isoform showed a two-fold higher expression in U4 flowers during drought than in the control variant. Ll-miR281/miR167 exhibited a two-fold decrease in U3 during drought compared to the control, which aligned with the expression pattern of FTRINITY_DN57891_c1_g1_i10 in this variant (Fig. 9d). This suggests that the miR167-*ARF6* regulatory axis may accelerate abscission under drought conditions. Additionally, transcripts encoding LlARF18 displayed differential expression between control and drought-stressed plants. FTRINITY_DN53543_c0_g2_i4 was expressed at lower levels in drought-stressed U1 and U3, whereas FTRINITY_DN56991_c0_g1_i8 showed higher accumulation in L3, U1, and U2. The *ARF18*-targeting Ll-miR333/miR160 was less abundant in drought variants of L3, L4, and U3, suggesting it may regulate *LlARF18*, particularly in L3 (Fig. 9e). These results indicate that the regulation of the auxin signal transduction pathway during drought stress is multifaceted and probably involves both miRNA-dependent and miRNA-independent mechanisms.

## Discussion

For decades, auxin has been recognised as a central regulator of virtually all plant processes, from ontogenesis to stress response, including flower development^4–8^ and abscission^49^. However, the detailed molecular network underlying its influence is gradually being elucidated through collaborative efforts. This study investigates the role of auxin in flower development and abscission, presenting new evidence on potential regulatory modules involved in these processes in yellow lupine (*Lupinus luteus* L.). Yellow lupine is not only a promising crop for enhancing food security in Europe but also an excellent model for studying flower abscission. Its developing flowers form 5-flower whorls along the inflorescence, which follows a basipetal pattern; the lower flowers develop earlier and have reduced competition for nutrients. Consequently, the lower whorls are preferentially retained on the plant, eventually forming fully developed pods. Auxin is a well-established regulator of nutrient sink formation, linking its function directly to the retention or abscission fate of flowers. However, the molecular networks determining flower fate prior to the formation of the abscission zone on the flower stalk remain poorly understood. By utilising yellow lupine as a model, we aim to provide more insights into these processes.

### Auxin regulates flower fate primarily *via* its signal transduction pathway

In our previous study^30^ we performed an experiment in which we removed almost all flowers from the yellow lupine inflorescence, excluding the last top whorl. This simple procedure resulted in the persistence of flowers on the plant and their development into pods, but only if it was performed for flowers up to stage 2A. This observation suggested that during the transition from stage 2 A to 2B, an irreversible determination of the flower fate occurs, probably caused by the shift of the nutrient sink. To confirm our previous RNA-Seq-based results strongly suggesting that auxin is involved in this process^30,50^, we performed IAA localisation in ovules. We observed slight differences in 2B stage ovules: in upper flowers, IAA displayed a slightly stronger signal in the outer integuments (Fig. 2a). This suggests auxin distribution may contribute to determining flower abscission.

To address this question, we conducted a field experiment. To determine whether the spatio-temporal pattern of IAA or or its perception and transduction are crucial for determining flower fate, we treated inflorescences with auxin (50 µM indole-3-acetic acid, IAA), or anti-auxins (100 µM N-1-naphthylphthalamic acid, NPA, or 100 µM p-chlorophenoxyisobutyric acid, PCIB). We selected two variants of inflorescence development – when flowers on the top (“top”) or bottom (“bottom”) whorls were at 2 A stage. Among the treatments, only the application of PCIB to the bottom whorls resulted in significant changes in the measured yield parameters (Fig. 2b, c). The lack of response to IAA or the blocker of auxin transport, NPA^41^, was unexpected, especially in case of the “bottom” variant – we expected that these treatments would lead to accelerated abscission of the bottom flowers. The studies published so far concerning the effect of auxin treatment on flowers focus on the abscission zone (e.g^7,51^.). In our experimental model, ectopic auxin distribution alone may not be sufficient for triggering abscission, implicating the involvement of additional factors. Given the fact that elements of the auxin signal transduction pathway are reported to play a role in flower abscission in a wide range of plant species (e.g^30,52,53^.), we investigated their role by PCIB treatments, which impairs the auxin signal transduction pathway by regulating Aux/IAA protein stability^40^. The increase in the retaining rate of generative organs after applying PCIB on the bottom whorls, when flowers at this site are 2 A stage, strongly suggests that the inhibition of the auxin signalling pathway elements expressed in the treated flowers inhibits or decreases the abscission. We conclude that auxin designates flower fate in closed flower buds primarily *via* its nuclear signalling pathway.

### miR393-TIR1/AFB, miR160-ARF17/18, and miR167-ARF6/8 are key auxin-related miRNA-target modules in yellow lupine flowers

Our detailed review of degradome data for yellow lupine flowers depicted a landscape of auxin-related miRNA-target modules, indicating miR393-TIR1/AFB, miR160-ARF17/18, and miR167-ARF6/8 are primarily active in the studied organs (Fig. 3). Interestingly, among the studied elements of the auxin signal transduction pathway, only *ARF6* encodes protein equipped in all the characteristic domains (Figs. 5, 6 and 7, Tables S4-5). However, ARF18 homologs in *Arabidopsis* naturally lack the PB1 domains and thus are independent of auxin because they cannot interact with Aux/IAA repressors^46^. In yellow lupine, their function is probably regulated in an auxin-independent manner. A big surprise was putative ARF8 proteins consisting only from the PB1 domains (Figs. 5 and 7). As their expression is relatively high in flowers, we did not exclude them from analyses. ARF proteins enable auxin to activate and deactivate genes and have their origins in *Charophytes*, the close ancestors of land plants. This origin arose from the rearrangement of single DBD, MD, and PB1 domains that could function independently^47,48^. On this basis, we hypothesise that their function is to modulate the dynamics of polymerisation with AuxIAA through their PB1 domains, and this way, weakening their repression independently from auxin.

The determination of specific functions carried out by individual nuclear auxin F-box receptors poses challenges due to their marked functional redundancy, as extensively shown through the analysis of *A. thaliana* knock-out mutants. In these plants, AFB1-3 homologues play a critical roles in flower development and are involved in endothecium lignification and anthesis^54^. Nevertheless, LlAFB3 appears to be the primary auxin receptor in yellow lupine flowers, as evidenced by its significantly higher expression level than other auxin receptors (Table S6).

In *A. thaliana AtARF6* and its paralogue *AtARF8*, regulate both stamen and gynoecium maturation. Flowers of *arf6 arf8* double-null mutant are unable to open and are distinguished by short petals, short stamen filaments, undehisced anthers, immature gynoecia^55^, aberrant vascular patterning, and lack of epidermal cell differentiation in petals^56^. Significantly, this phenotype is affected by *AtARF6* and *AtARF8* gene dosage quantitatively^55^. We suppose the roles of LlARF6 and LlARF8 are similar, i.e. they control the time-correlated achievement of milestones in flower development. It is striking that no canonical form of LlARF8 was identified, and in yellow lupine flowers, these proteins consist only of the type II PB1 domain (Figs. 5c and 7a). ARFs with only one PB1 type I or type II domain could potentially diminish the ARF-Aux/IAA interaction by creating “blunt ends” in the PB1 polymer, thereby influencing the kinetics of auxin signal transduction^57^. This hypothesis requires more evidence.

ARF18 also has a documented but scarce role in flower development. In roses, ARF18 interacts with Histone Deacetylase 6 to regulate flower evocation^58^. Later, during stamen development, its transcription is controlled by RhAGL24^59^. It is also known that *ARF18* is downregulated in yellow lupine roots^60^, which indicates its transcriptional responsiveness to this stress. However, evidence for its involvement in flower fate is lacking. The decrease in LlARF18-encoding FTRINITY_DN56991_c0_g1_i8 accumulation in upper stage 2 and lower stage 4 flowers (Fig. 9e) indicates the role of *ARF18* expression dynamics in flower fate and suggests that higher levels of this transcription factor are required till the stage 3 to maintain the flower on the inflorescence. The increase in Ll-miR333/miR160 accumulation at the time of anthesis and its subsequent increase (Fig. 9e) suggests its involvement in post-anthesis processes, like fertilisation or elongation of the ovary wall after the fruit set.

### ***LlAFB3***, ***LlARF6***, and ***LlARF18*** respond to drought stress and may be modulated by miR393, miR167, and miR160

The accumulation patterns of selected auxin-related transcripts in flowers during drought provide valuable insights into the potential role of nuclear auxin signaling in stress response. In upper flowers (U2-4), we propose an increase in auxin sensitivity, driven by the elevated expression of *LlAFB3* (Fig. 9c). However, literature on TIR1/AFB proteins in other species reveals inconsistent roles, suggesting species-specific functions. For instance, in strawberry, *FveAFB5* positively influences fruit development, thereby not promoting abscission^61^. Conversely, in tomato, overexpression of plum *PslTIR1* reduces flower number and alters morphology^62^. The absence of a clearly opposing miRNA–target accumulation pattern suggests that *LlAFB3* is primarily regulated at the transcriptional level, with *Ll-miR224/miR393* likely targeting other transcripts for downregulation.

The increased expression of *LlARF6* during drought in upper flowers, particularly the FTRINITY_DN57891_c1_g1_i1 isoform in U4 flowers, suggests a role in facilitating accelerated flower abscission under this stress. Surprisingly, Ll-miR281/miR167 does not exhibit differential accumulation in this variant, indicating that it likely does not mediate drought responses through this axis. However, its accumulation decreases markedly in the U3 variant, exhibiting a negative correlation with the FTRINITY_DN57891_c1_g1_i10 isoform. Nonetheless, this difference appears minimal and may be limited to a very specific region of the organ (Fig. 9d). Alternatively, the main regulation of its expression may occur on the transcription level, e.g. via *DREB2* homologue^63^.

LlARF18 appears to play a role in the response of flowers to drought. Both analysed transcripts, FTRINITY_DN53543_c0_g2_i4 and FTRINITY_DN56991_c0_g1_i8, as well as *ARF18*-targeting Ll-miR333/miR160, were differentially expressed in stressed flowers. The accumulation pattern suggests the miRNA-mediated regulation of *ARF18* is particularly significant in L3 flowers (Fig. 9e). Little is known about the role of the studied module in stress. ARF18 is known to regulate root development during stress in lotus^64^. In peanut, miR160-*ARF18* module is involved in response to salt stress^65^. In banana, this module is involved in the adaptation to potassium stress^66^. Our findings extend these insights, highlighting its involvement in the drought stress response of post-anthesis yellow lupine flowers.

In many studies examining miRNA-target expression, explicit reverse correlations are not always observed (see e.g^67,68^.). The lack of clear correlation between miRNAs and their degradome-confirmed targets in yellow lupine flowers suggests that the regulation of the auxin signal transduction pathway during drought stress is multifaceted, likely involving both miRNA-dependent and miRNA-independent mechanisms, such as transcriptional regulation. Despite this complexity, the differential expression of miRNAs and their auxin-related targets indicates their involvement in development and drought stress as a part of a complex regulatory network rather than as the dominating factor. Nevertheless, our findings provide valuable insights into the potential roles of auxin-related genes in flower development, abscission and drought response in yellow lupine, paving the way for future experiments to validate these observations.

## Conclusions

The successful development of flowers is vital for the transmission of genes to subsequent generations and, ultimately, the survival of the species. During stresses, especially droughts, this process is threatened by excessive flower abscission. Our results indicate that canonical auxin-related miRNA-transcript modules play a role in yellow lupine flower development and determine flower fate before abscission zone formation. These regulatory molecules are also involved in drought stress response in flowers. Our study advances the understanding of the complex mechanisms underlying flower development and auxin signalling in yellow lupine, providing knowledge that can be used to foster innovations leading to more productive and resistant lupine varieties enhancing food safety in Europe. However, further studies, such as those employing reverse genetics and interaction assays, are needed to fully elucidate the investigated processes.

## Supplementary Information

Below is the link to the electronic supplementary material.

Supplementary Material 1

Supplementary Material 2

## Data Availability

Some raw data essential for interpretation are provided as Supplementary Files. The remaining raw data and the Python and R scripts are available upon request by contacting Milena Kulasek (milena.kulasek@umk.pl).
