# Supplementary material for: miRNA-targeted auxin nuclear signalling elements orchestrate flower fate and drought response in yellow lupine
Source: Sci Rep. 2025 Nov 10;15:39273. doi: 10.1038/s41598-025-22989-x (PMC12603313; doi:10.1038/s41598-025-22989-x)
Supplement: Supplementary file 2 — Supplementary Material 2 [file 41598_2025_22989_MOESM2_ESM.pdf]

Supplementary Material (Figures S1-S9)

miRNA-Targeted Auxin Nuclear Signalling Elements Orchestrate Flower Fate and Drought Response in Yellow Lupine

Milena Kulasek [1,2]*, Paulina Glazińska [2,3]

[1] Department of Genetics, Faculty of Biological and Veterinary Sciences, Nicolaus Copernicus University in Torun, Poland

[2] Centre for Modern Interdisciplinary Technologies, Nicolaus Copernicus University in Torun, Poland

[3] Department of Plant Physiology and Biotechnology, Faculty of Biological and Veterinary Sciences, Nicolaus Copernicus University in Torun, Poland

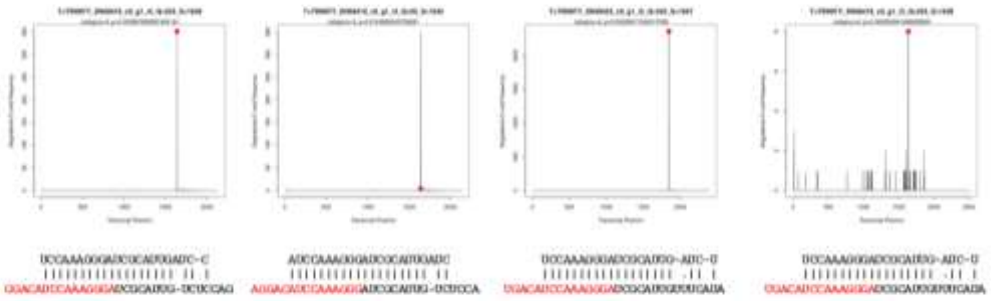

Figure S1. Identification of miRNA-directed cleavage sites in *LlAFB3*- and *LlTIR*-encoding transcripts. T-plots showing the predicted cleavage sites for miRNA-mRNA pairs confirmed in degradome-Seq data. Alignments below show the comparisons of miRNAs (upper sequence) and the reverse complementary fragment of the target site (lower sequence). The end of the red sequence is the cleavage site.

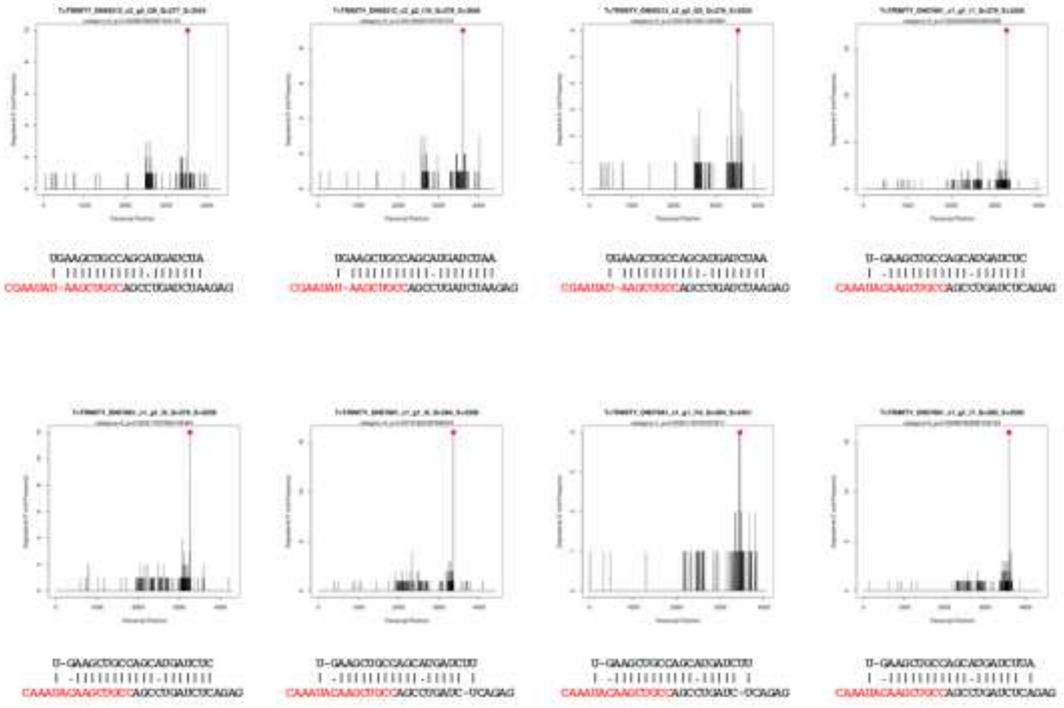

Figure S2. Identification of miRNA-directed cleavage sites in *LlARF6/8*-encoding transcripts. T-plots showing the predicted cleavage sites for miRNA-mRNA pairs confirmed in degradome-Seq data. Alignments below show the comparisons of miRNAs (upper sequence) and the reverse complementary fragment of the target site (lower sequence). The end of the red sequence is the cleavage site.

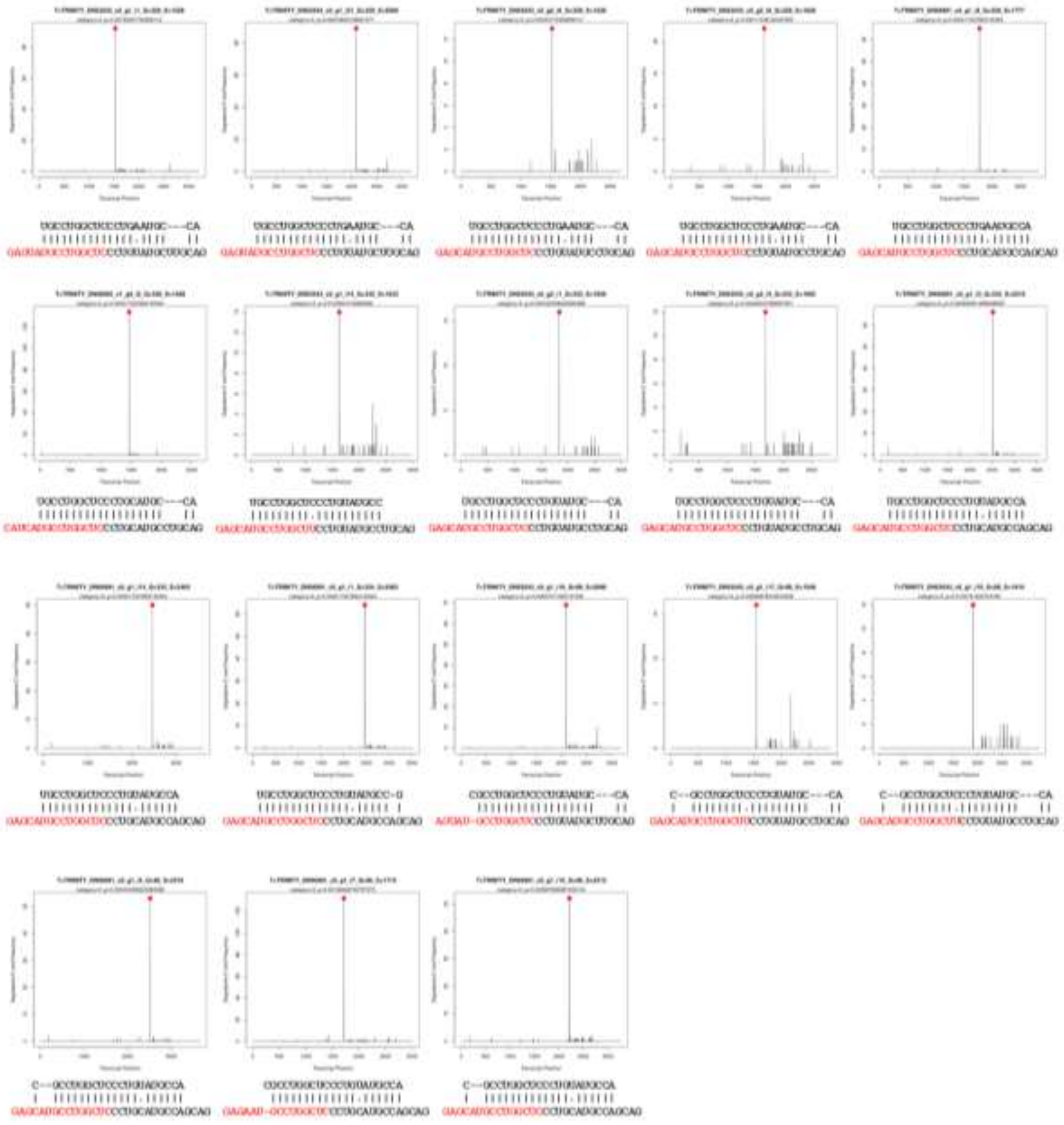

Figure S3. Identification of miRNA-directed cleavage sites in *LlARF17/18*-encoding transcripts. T-plots showing the predicted cleavage sites for miRNA-mRNA pairs confirmed in degradome-Seq data. Alignments below show the comparisons of miRNAs (upper sequence) and the reverse complementary fragment of the target site (lower sequence). The end of the red sequence is the cleavage site.

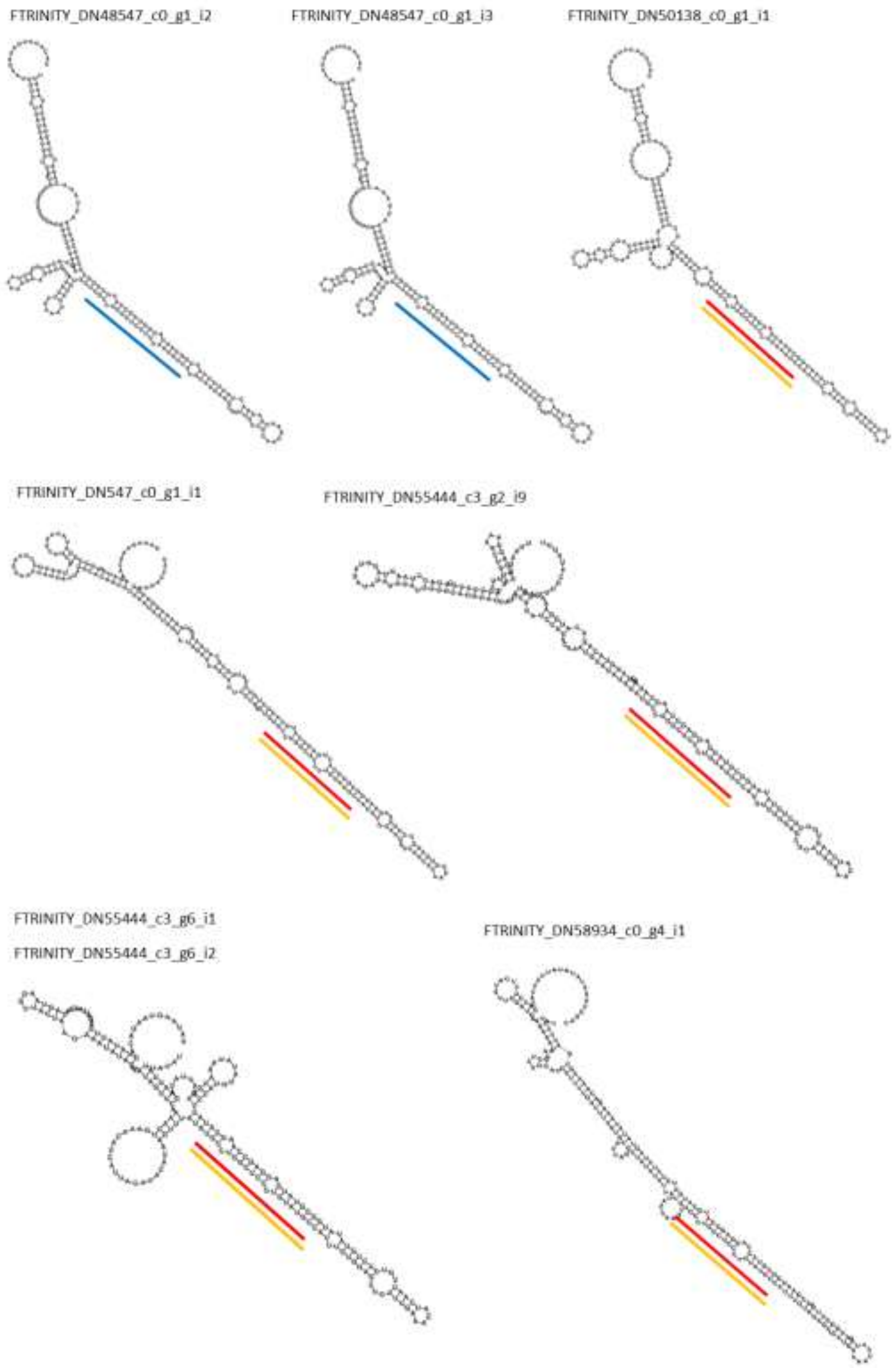

Figure S4. Secondary structures of 100 nt-[mature miRNA sequence]-100 nt fragments of transcripts identified as possible precursors of miRNAs belonging to the MIR160 family. Blue bars indicate Ll-miR329 sequence, red – Ll-miR332, and yellow – Ll-miR333.

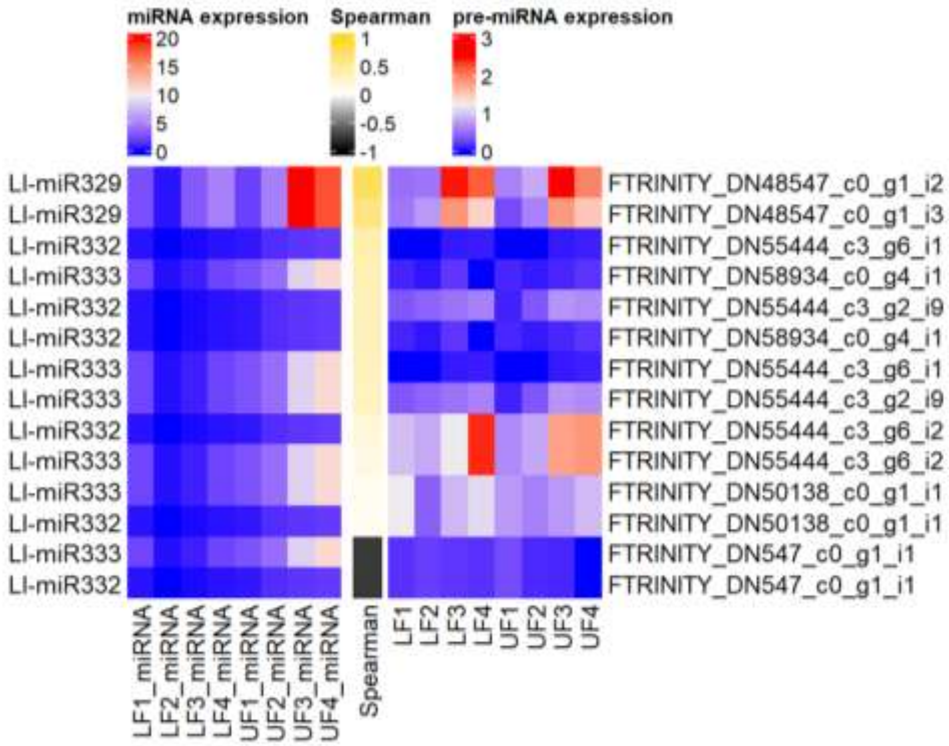

Figure S5. Correlation of expression levels of miRNAs belonging to the MIR160 family and transcripts identified as their possible precursors.

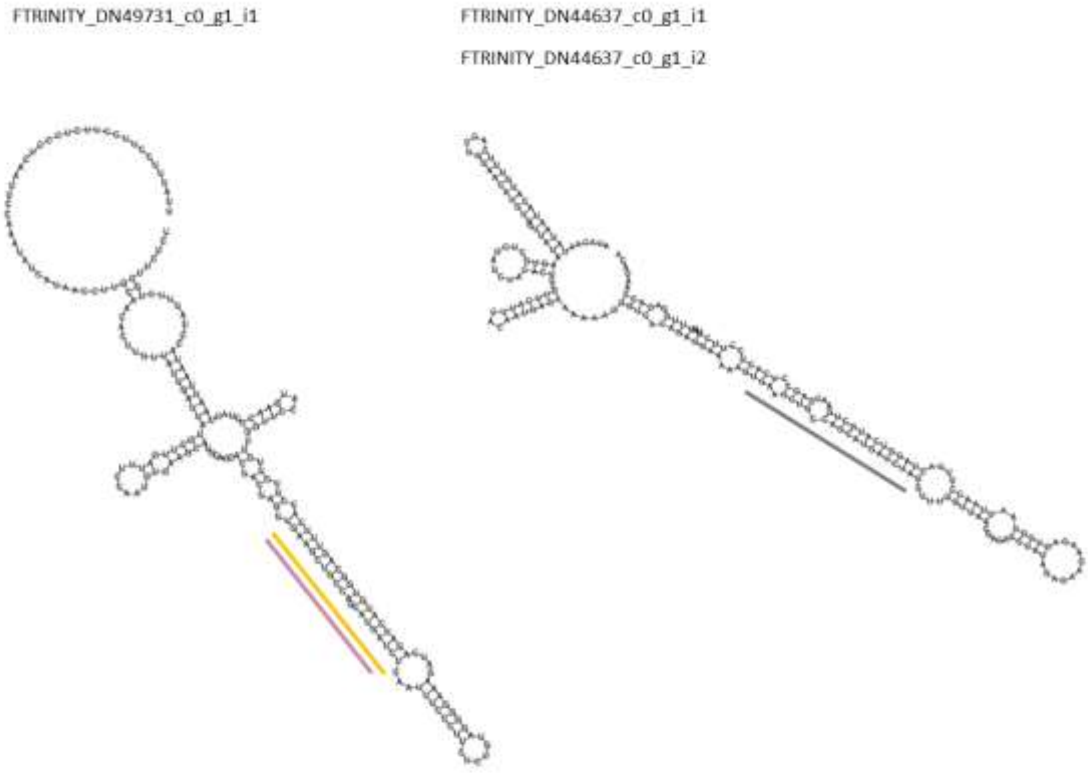

Figure S6. Secondary structures of 100 nt-[mature miRNA sequence]-100 nt fragments of transcripts identified as possible precursors of miRNAs belonging to the MIR167 family. Gray bar indicates Ll-miR277 sequence, violet – Ll-miR284, and yellow – Ll-miR285.

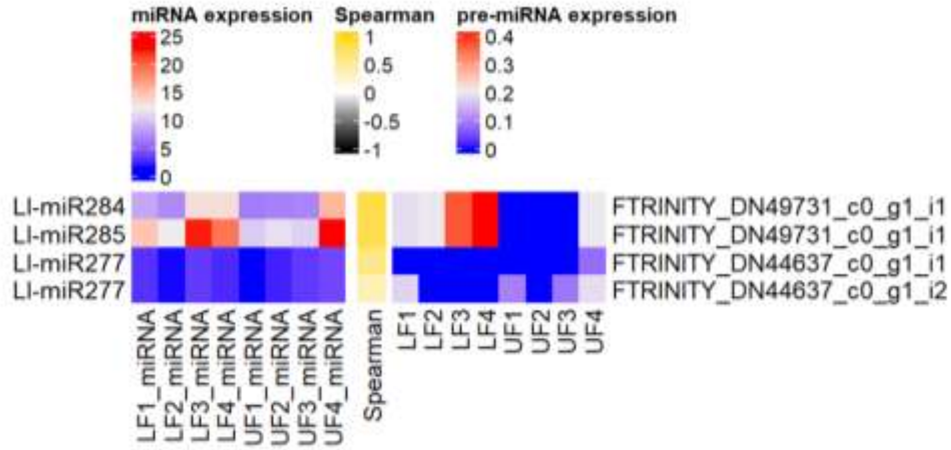

Figure S7. Correlation of expression levels of miRNAs belonging to the MIR167 family and transcripts identified as their possible precursors.

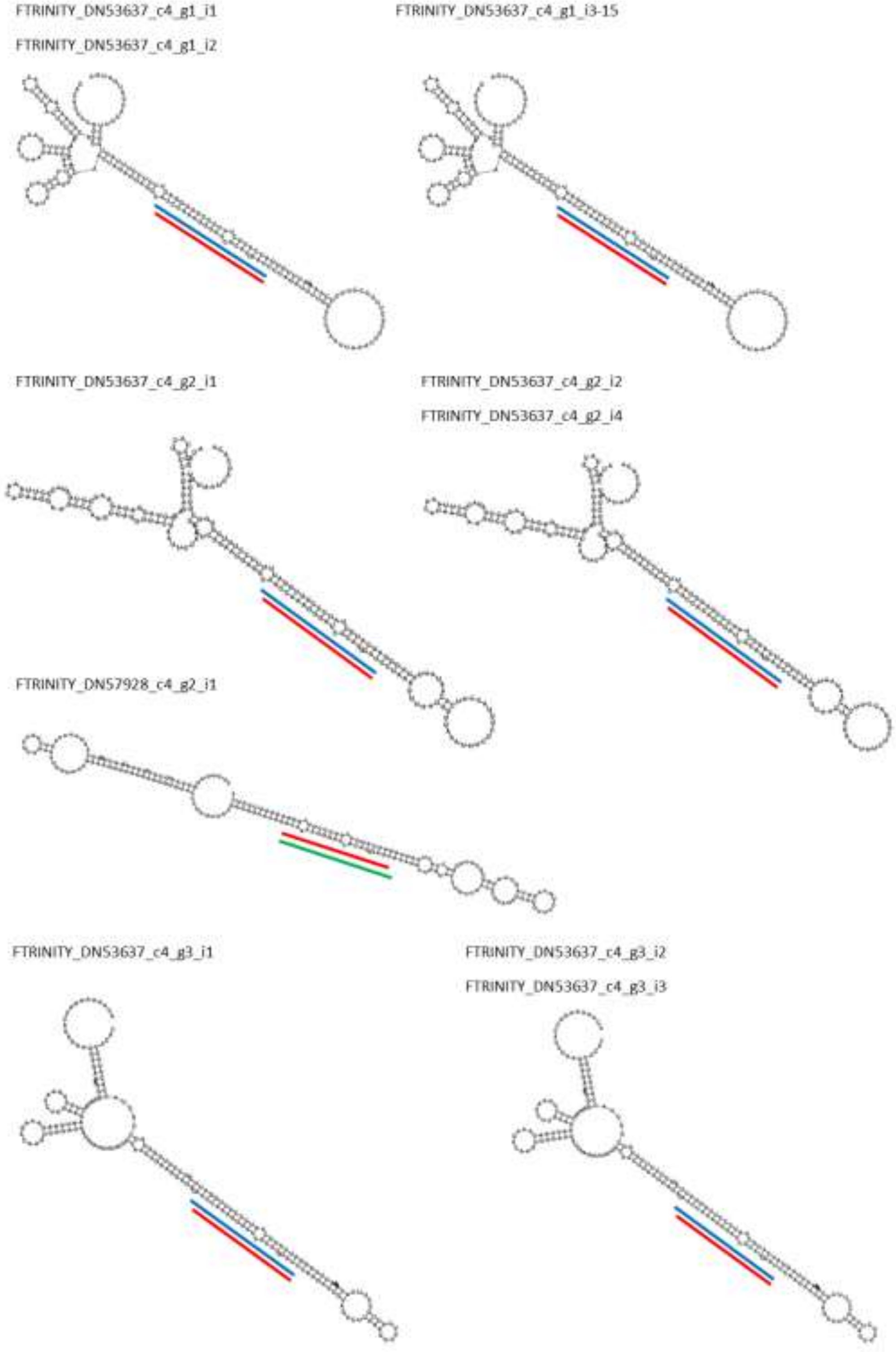

Figure S8. Secondary structures of 100 nt-[mature miRNA sequence]-100 nt fragments of transcripts identified as possible precursors of miRNAs belonging to the MIR393 family. Blue bars indicate Ll-miR55 sequence, red – Ll-miR223, and green – Ll-miR225.

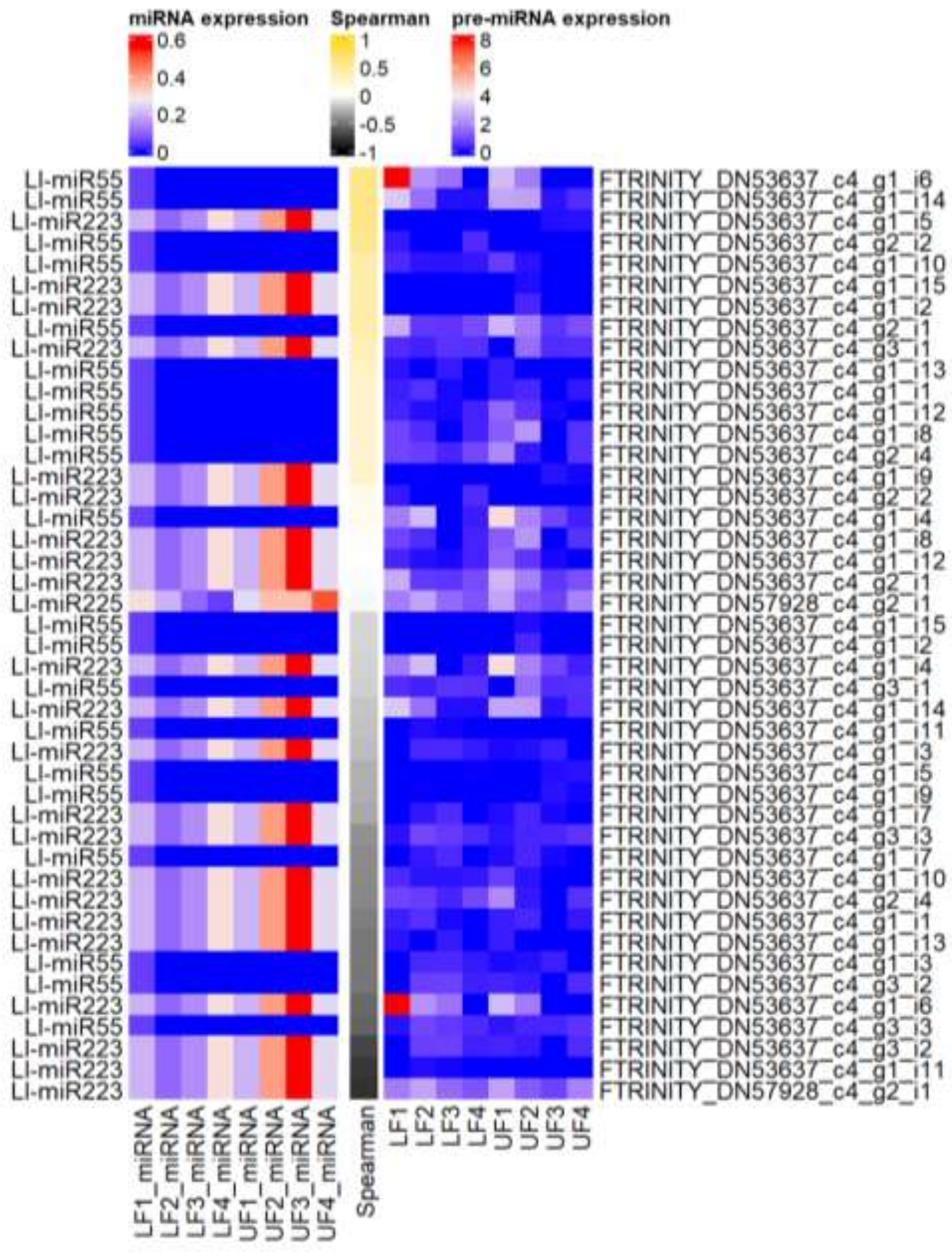

Figure S9. Correlation of expression levels of miRNAs belonging to the MIR393 family and transcripts identified as their possible precursors.
